# Hidden MODY—Looking for a Needle in a Haystack

**DOI:** 10.3389/fendo.2018.00355

**Published:** 2018-07-02

**Authors:** Jana Urbanová, Ludmila Brunerová, Jan Brož

**Affiliations:** ^1^Second Department of Internal Medicine, Third Faculty of Medicine, Center for Research of Diabetes, Metabolism and Nutrition, University Hospital Královské Vinohrady, Prague, Czechia; ^2^Department of Internal Medicine, Second Faculty of Medicine, Charles University, Prague, Czechia

**Keywords:** MODY, *GCK*-MODY, *HNF1A*-MODY, *HNF4A*-MODY, diagnostics, differential diagnosis

## Introduction

MODY (Maturity onset diabetes of the young) is a specific type of diabetes caused by mutation in a single gene, involved in the development and function of the β-cells, inherited in an autosomal dominant manner (1). Out of fourteen, up to date discovered, MODY genes (2) the most often affected ones include *GCK* (gene encoding glucokinase enzyme) and *HNF1A* (encoding the transcription factor—hepatocyte nuclear factor 1α), which altogether account for approximately 80% of all MODY cases (3). Mutations in other genes (e.g., *HNF4A* or *HNF1B*—hepatocyte nuclear factor 4α and 1β), occur rarely (4).

Although MODY represents a rather scarce diabetes type (1), searching for MODY among much more prevalent forms of diabetes is important and desirable for its clear impact on clinical practice—for appropriate diabetes management with the most suitable treatment (accompanied with improved quality of life) (5, 6), for assessing the real risk of development and progression of specific diabetic complications in each MODY type, as well as for early diagnosis in the patient's relatives and offspring.

Nevertheless, overwhelming majority of MODY patients worldwide remains misdiagnosed (3, 7). Moreover, no unitary and up-to-date diagnostic guidelines have been established so far, and also it is not obvious, which approach to correct identification of MODY patients is optimal.

The aim of this communication is to present our experiences with searching for patients with MODY in the context of current diagnostic proceedings and actual study outputs available.

## How to recognize MODY among diabetic patients?

MODY is traditionally characterized by the following features (8):

- early onset of diabetes (i.e., <25 years of age);
- insulin-independence (i.e., preserved endogenous insulin secretion >5 years of diabetes duration);
- and autosomal dominant inheritance (i.e., family history of glycaemic disorders at least in two consecutive generations).

Thus, a young patient diagnosed with diabetes before the age of 25 years with a marked family history of diabetes in one parental line and signs of ongoing insulin secretion (the absence of ketoacidosis/DKA at the time of diabetes onset and later on /under circumstances significantly increasing its risk or detectable C-peptide for years and obviously low doses of insulin required) should be suspected of MODY and recommended to molecular genetic testing (8); especially when islet autoimmunity and insulin resistance are not simultaneously present, so the patient doesn't fit either to type 1 or 2 type diabetes.

## Clinical characteristics of MODY

*GCK*-MODY is suspected in patients diagnosed with mild and asymptomatic fasting hyperglycaemia (typically 5.4–8.3 mmol/L) with relatively low glycated hemoglobin/HbA1c level (ranging usually 40–60 mmol/mol) (8, 9). In most cases it is detected incidentally during routine blood tests in adolescence or adulthood (although hyperglycaemia is present already from birth) (10). In younger age, however, the most prevalent type 1 diabetes (11) should be excluded; whereas the likelihood of type 2 diabetes grows with older age or obesity. In our experience, suspicion of *GCK-*MODY in contrast to type 1 diabetes at the time of diagnosis increases in the absence of DKA and islet autoantibodies, normal level of fasting C-peptide and presence of diabetes in one parent, whereas in contrast to type 2 diabetes *GCK-*MODY is suspected in rather normal body mass index/BMI, the absence of insulin resistance (e.g., dyslipidemia, hypertension) or modest rise in 2-h glucose level on oGTT/oral glucose tolerance test (< 4.6 mmol/L in 95% of cases, 12). On the contrary, signs that point out to *GCK*-MODY in patients with already established diagnosis of diabetes (labeled falsely as type 1 or type 2), in our opinion, include non-progressive character of glycaemic disorder (relatively stable glycaemia and HbA1c), non-decreasing (normal) endogenous insulin secretion, low efficacy of oral antidiabetics (e.g., metformin) administered or relatively low doses of insulin required and absence of vascular complications after many years of diabetes duration. Clinical picture of *GCK*-MODY could be, however, modified in some adult patients by their genetic background (e.g., polymorphisms in other diabetes-related genes), which could accentuate some features more typical for type 2 diabetes.

*HNF1A*-MODY is characterized by progressive glucose dysregulation, clinically presented at first with postprandial, later fasting hyperglycaemia, usually during adolescence or early adulthood (1, 8); thus, a large glucose increment of > 5 mmol/L can be observed on oGTT (12) in the early stages of the disease, whereas fasting blood glucose levels remain normal (14). Due to low renal threshold for glucose, glycosuria following a carbohydrate load often occurs, even before the clinical features of diabetes become apparent (14). Individuals with *HNF1A*-MODY similarly to other patients with diabetes can present with typical hyperglycaemia-related symptoms as polyuria, polydipsia and weight loss. Early signs that make diagnosis of *HNF1A*-MODY (in contrast to type 1 diabetes) possible include family history of diabetes, manifestation of diabetes without DKA, absence of islet antibodies, detectable C-peptide levels; similarly, younger age <30 years (11), lower BMI, presence of hypertension and triglycerides (15), higher HDL-cholesterol (>1.3 mmol/L) (16), eventually non-elevated fasting C-peptide level and marked sensitivity to SUR/sulphonylurea derivatives (5) are more indicative for *HNF1A*-MODY compared to type 2 diabetes.

In our opinion, level of fasting C-peptide measured at the time of diabetes manifestation is the most beneficial in discriminating MODY from type 1 diabetes if detected either very low (<150 pmol/L)—suggesting type 1 diabetes, or within normal range (300–600 pmol/L)—favoring MODY. Normal C-peptide, however, needn't necessarily exclude type 1 diabetes since endogenous insulin production in type 1 diabetes may be detectable for a variable length of time, including honeymoon period (17), particularly in LADA (late-onset autoimmune diabetes in adults). Thus, in case of uncertainty, to differentiate type 1 diabetes in the early stages we prefer the assessment of stimulated (post-meal) C-peptide (with levels >200 pmol/L showing to MODY as described (18, 26). Persistence of fasting C-peptide production 3–5 years after diagnosis further increases suspicion of MODY (26). On the contrary, we consider the detection of islet autoantibodies most useful soon after the diabetes diagnosis, as their prevalence in type 1 diabetes declines with the duration of the disease (19), whereas, in our experience, they could be detectable in some MODY patients even more than a decade after the diabetes onset, particularly in patients with poor glycaemic control (20).

Although significantly lower hsCRP level has been identified as the sign discriminating the patients with *HNF1A*-MODY from those with type 2 diabetes (21), we did not find this parameter useful in our clinical experience. Furthermore, some recent studies (comparing *HNF1A*-MODY to young-onset type 2 diabetes) challenged this paradigm, indicating that hsCRP does not improve diagnosis (7, 22). Moreover, hsCRP is not useful if elevated (>10 mg/L) as this usually indicates the presence of confounding inflammation, and should be repeated after a few weeks (23).

*HNF4A*-MODY shares similar clinical characteristic with *HNF1A*-MODY, except for the low renal threshold and possibly later diagnosis (24). We consider this type of MODY the most difficult to distinguish from type 2 diabetes, since *HNF4A*-MODY patients at the diagnosis may be older and may present with dyslipidaemia similar to patients with type 2 diabetes, particularly higher LDL- and lower HDL-cholesterol (4, 24). Differential diagnosis may facilitate higher birth weight (>4.4 kg at term), fetal macrosomy (56% of *HNF4A*-MODY individuals) and history of transient neonatal hyperinsulinaemic hypoglycaemia (15% of cases) (25).

## Obstacles in searching for MODY

Despite well-defined clinical presentation of MODY, reliability on clinical features alone seems insufficient in identification of MODY among other and more common types of diabetes. Less than 50% of all MODY cases actually meet traditional features of MODY (26). Moreover, clinical heterogeneity of MODY and possible overlapping clinical characteristics with type 1 and type 2 diabetes make the correct identification of MODY an uneasy task.

In accordance with Shields et al. (11) we consider having a parent with diabetes to be a very strong predictor of MODY compared with type 1 diabetes (increasing the probability of MODY by 23 times in those initially classified as type 1 diabetes). Though, in patients with MODY and type 2 diabetes, a positive family history of diabetes is reported in the same frequency (26, 27). On the other hand, the absence of diabetes in family members does not exclude MODY. Recent study has revealed much higher frequency of spontaneous de-novo mutations in *GCK, HNF1A* and *HNF4A* genes than previously assumed (28); moreover in *GCK*-MODY, discrete fasting hyperglycaemia may not be yet recognized in a parent, thus, family history could be seemingly negative. In a major part of our MODY patients at least one family member (parent, sibling) was identified. The only exception represents patients with *HNF1B*-MODY (in whom de-novo mutations occur more often) or accidental cases with unknown or prematurely dead relatives, eventually with so far unexamined glycaemia in parents.

Variable age at the time of diabetes onset, which is actually determined by many factors [e.g., the type, position and penetration of causal mutation in *HNF1A* and *HNF4A* genes (29), presence of risk polygenic single nucleotide polymorphisms of type 2 diabetes (30) or exposure to hyperglycaemia *in utero* (13)] represents another problem in differential diagnosis. For example, high penetrance of *HNF1A* mutation confers development of diabetes before 25 years of age in only 63% mutation carriers, but before the age of 35 in nearly 80% of individuals (4), whereas variable penetrance of *HNF4A* mutations cause that some of their carriers do not develop diabetes until the fourth decade of life (18). Thus, in patients with *HNF1A*/*HNF4A* mutation, diabetes may present later than before traditionally defined 25 years of age. Conversely, proportion of *GCK*-MODY in older populations (>35 years) is low and mild hyperglycaemia in adults is more likely to reflect type 2 diabetes than *GCK*-MODY (26, 31).

Due to globally increasing prevalence of obesity, expected non-obese habitus and absence of insulin resistance needn't necessarily apply to all MODY cases. Overweight, obesity and insulin resistance seem to be underestimated among MODY patients, at least in some populations (32, 33). However, in our cohort of patients referred for molecular genetic testing, BMI was usually just below the normal range among those with confirmed mutation in one of the MODY gene, probably because of the pre-selection of lean individuals for the testing. Nevertheless, in our own clinical practice, particularly overweight occurs more frequently in the older age groups.

Conflicting data exists on autoantibody positivity in MODY patients. Contrary to originally described low prevalence of islet autoantibodies in MODY (34), our own clinical experience (20), similarly to other authors (26, 35) shows that the prevalence of autoantibodies (especially GADA) in patients with confirmed MODY mutations is significantly higher. Thus, we fully agree with the recommendation that in the case of strong clinical suspicion, the presence of islet autoantibodies should not preclude genetic testing (23). Another diagnostic pitfall is the presence of DKA (36, 37).

Since strict adherence to traditional clinical criteria is insufficient in identifying MODY patients, other algorithms (10, 23, 26, 38), predictive models (11) or selected biomarkers (39) have been tested. Widening of the clinical criteria for genetic testing beyond current guidelines (to all patients diagnosed up to the age of 30 years, regardless of the family history of diabetes, GADA positivity, or insulin resistance) almost doubled the number of MODY diagnoses (26). We strongly recommend using Shield's predictive model (11), particularly in case of clinical uncertainty whether to indicate molecular genetic testing or not. Finally, biomarker-based screening pathway (combining assessment of endogenous insulin secretion with measurement of GADA and IA2A) increased the discriminatory ability and facilitated identification of even atypical cases and rarer forms of monogenic diabetes, which traditional criteria may miss (39) although as we have just pointed out the results obtained should be interpreted with caution. In Table 1, we tried to summarize typical clinical and laboratory features, together with some diagnostic pitfalls discussed above.

**Table 1:** Rational approach to identify MODY patients—gradual evaluation of routinely available clinical and laboratory data increasing suspicion of MODY.

	***GCK*-MODY**	***HNF1A*-MODY**	***HNF4A*-MODY**
**NEW PATIENT DIAGNOSED WITH HYPERGLYCAEMIA**			
Age at diagnosis	Variable (hyperglycaemia present since birth may be diagnosed at any time, usually incidentally)	Adolescence or early adulthood (~80% before the age of 35)	Variable—adolescence or adulthood
Family history	Usually positive, a parent with mild fasting hyperglycaemia	Positive usually at least in two consecutive generations, diabetes of any type diagnosed usually at early age	
Symptoms	Asymptomatic	Usually polyuria, polydipsia, tiredness etc., absence of DKA	
Nutritional status	Usually lean, obesity within population frequency			
Glycaemia (mmol/L) FPG 2 h glucose increment on oGTT	Mild (5.4–8.3) < 3.0	Normal-increased FPG with marked hyperglycaemia postprandially; high FPG and PPG >5.0	
HbA1c (mmol/mol)	Near normal (38–56 if aged ≤ 40 years; 41–60 if >40 years)	Variable	
C-peptide Fasting Stimulated (pmol/L)	Preserved 100–900	Preserved 100–700	
Islet autoantibodies	Usually negative		
Other features	Usually absence of insulin resistance (e.g., dyslipidemia, hypertension)		Low HDL cholesterol and elevated LDL cholesterol, but low triglycerides	
	–	Normal/raised HDL cholesterol (>1.3 mmol/L) Glycosuria at blood glucose < 10.0 mmol/L	Fetal macrosomia, higher birth weight (>4.4 kg at term) and congenital hypoglycaemic hyperinsulinaemia in some cases
Treatment outcomes	Low efficacy of oral antidiabetics (e.g., metformin) or insulin doses requirements	Marked sensitivity to SUR with increased risk of iatrogenic hypoglycaemia, low insulin doses requirements (usually < 0.3 IU/kg)	
**PATIENT LABELED YEARS WITH TYPE 1 OR TYPE 2 DIABETES**			
Clinical course of diabetes	Non-progressive, stable glycaemia and HbA1c level	Progressive	
Chronic diabetes complications	No microvascular and macrovascular complications detected	Rapid progression of microvascular and macrovascular complications in poorly controlled patients	
Acute diabetes complications	Absence of ketoacidosis e.g., in missed or inadequate insulin treatment, illness or other risk situations		
	–	Increased risk of iatrogenic hypoglycaemia in SUR treated patients	
Fasting (or random) C-peptide (pmol/L)	Persistent production (exceeding honeymoon period and >5 years postdiagnosis)		

## Conclusion

Substantial heterogeneity and overlapping in clinical features of MODY with other common types of diabetes considerably contribute to omission and misclassification of MODY in routine clinical praxis. Currently no uniform diagnostic algorithm exists and it is not evident which approach is optimal in identification of MODY patients. A comprehensive evaluation of patient history, disease manifestation and progression, treatment outcomes and routine laboratory parameters (considering the possible exceptions listed above) represents, in our experience, a rational approach in recognizing new patients with MODY and also individuals scattered for many years between patients with other types of diabetes.

## Author contributions

JU prepared the draft of the paper, LB and JB revisited it critically for important intellectual content.

### Conflict of interest statement

The authors declare that the research was conducted in the absence of any commercial or financial relationships that could be construed as a potential conflict of interest.

## Abbreviations

BMI: body mass index
DKA: diabetic ketoacidosis
GADA: autoantibodies to glutamic acid decarboxylase
GCK: glucokinase
HbA1c: glycated haemoglobin
HDL: high density lipoprotein
HNF1A: hepatocyte nuclear factor 1 alpha
HNF1B: hepatocyte nuclear factor 1 beta
HNF4A: hepatocyte nuclear factor 4 alpha
hsCRP: high sensitive C-reactive protein
IA2A: autoantibodies to IA-2
LADA: Late-onset Autoimmune Diabetes in Adults
LDL: low density lipoprotein
MODY: Maturity Onset Diabetes of the Young
OGTT: oral glucose tolerance test
SUR: sulphonylurea derivatives.
